# Sex bias in basic and preclinical age-related hearing loss research

**DOI:** 10.1186/s13293-018-0185-7

**Published:** 2018-06-13

**Authors:** Dillan F. Villavisanis, Katrina M. Schrode, Amanda M. Lauer

**Affiliations:** Department of Otolaryngology—Head and Neck Surgery, Center for Hearing and Balance, Johns Hopkins University School of Medicine, 720 Rutland Ave., Baltimore, MD 21205 USA

**Keywords:** Sex bias, Hearing loss, Basic, Preclinical, Animals, NIH

## Abstract

**Objectives:**

This study aims to determine if there is sex bias in basic and preclinical research on age-related hearing loss for the 10-year period of 2006–2015, prior to the NIH mandate of including sex as a biological variable in 2016.

**Design:**

Manuscripts were identified in PubMed for the query “age-related hearing loss” for the 10-year period of 2006 to 2015. Manuscripts were included if they were original research (not reviews or meta-analyses), written in English, contained an abstract, used animals, and were primarily on age-related hearing loss. These criteria yielded 231 unique manuscripts for inclusion in the study analysis. The text of each manuscript was screened for the sex of the animals, the number of male and female animals, the discussion of sex-based results, the study site (US or international), and the year of publication.

**Results:**

Only two thirds of manuscripts reported the sex of animals used in the experiments, and of these, 54% used both sexes, 34% used males only, and 13% used females only. In papers reporting sex and number of animals used, 67% were males and 33% were females. Over twice as many internationally based studies used males only compared to US-based studies. Only 15% of all manuscripts discussed sex-based results.

**Conclusions:**

Sex bias is present in basic and preclinical age-related hearing loss research for the manuscripts screened in the 10-year period. Equal inclusion of both males and females in basic and preclinical age-related hearing loss research is critical for understanding sex-based differences in mechanisms and for effective treatment options.

## Background

Age-related hearing loss (ARHL) or presbycusis is the gradual loss of hearing sensitivity with age. It affects one in three people between the ages of 65 to 74 and nearly half of people over 75 in the USA (National Institute on Deafness and Other Communication Disorders). Since the exact biological causes and mechanisms of ARHL are not completely known [1], preclinical and basic science research has been crucial for informing the current understanding of ARHL through physiological [2], anatomical [3], behavioral [4], and genetic [5] studies. ARHL has clinical associations with cognitive decline [6], social isolation [7], and memory loss [8], as well as increased risk of falls [9], hospitalization [10], and mortality [11], which underscore the importance of preclinical and basic science investigations of this condition. There are well-known differences in the trajectory of ARHL for men and women; studies indicate that men experience a faster decline in hearing ability than women [12, 13].

Using animals in preclinical and basic science research provides a unique advantage in understanding the effects of age on hearing loss since the auditory environment and other potentially interacting factors can be carefully controlled. However, the number of preclinical studies that explicitly investigated the effects of sex-based differences on AHRL is relatively small [14–20], and further investigations are needed for a more comprehensive understanding. In C57BL/6 mice, females demonstrated accelerated increases in auditory brainstem response (ABR) thresholds with age compared to males [19, 20]. In another study, young male and female CBA mice (substrain unspecified) demonstrated similar distortion product otoacoustic emissions (DPOAEs), but females demonstrated higher premenopausal DPOAE levels, which declined postmenopause. Old CBA females also had lower ABR thresholds than old CBA males [18]. Studies investigating the protective effects of exposure to an augmented acoustic environment against ARHL have also shown differences in treatment effectiveness between males and females [15, 16, 20].

Basic and preclinical research has clear and direct implications for translational research and clinical outcomes [21]. Therefore, an understanding of sex as a biological variable and its effects on preclinical research, and here particularly on ARHL research, is important for informing treatment and prevention strategies. The National Institutes of Health (NIH) has required the inclusion of men and women in NIH-funded clinical research since 1993 through the Revitalization Act, but the inclusion of both sexes in basic and preclinical research was not required by the NIH until January of 2016 [22]. This is significant as NIH-funded basic research contributes substantially to drug development [23], and studies have demonstrated that mammalian traits in both wildtypes and mutants are influenced by sex [24].

Other fields have addressed the issue of sex bias in preclinical and basic research including dermatology [25], cardiology [26], surgery, [27], and general neuroscience [28, 29]. However, sex bias has only been addressed in one study with a focus on auditory neuroscience that identified sex bias in preclinical and basic noise-induced hearing loss (NIHL) research [30]. In the present study, the representation of sex as a biological variable in basic and preclinical studies of ARHL was evaluated for the 10-year period from January 2006 to December 2015 to understand sex bias in studies of ARHL prior to the NIH mandate.

## Materials and methods

### Literature search and inclusion criteria

**Fig. 1:**
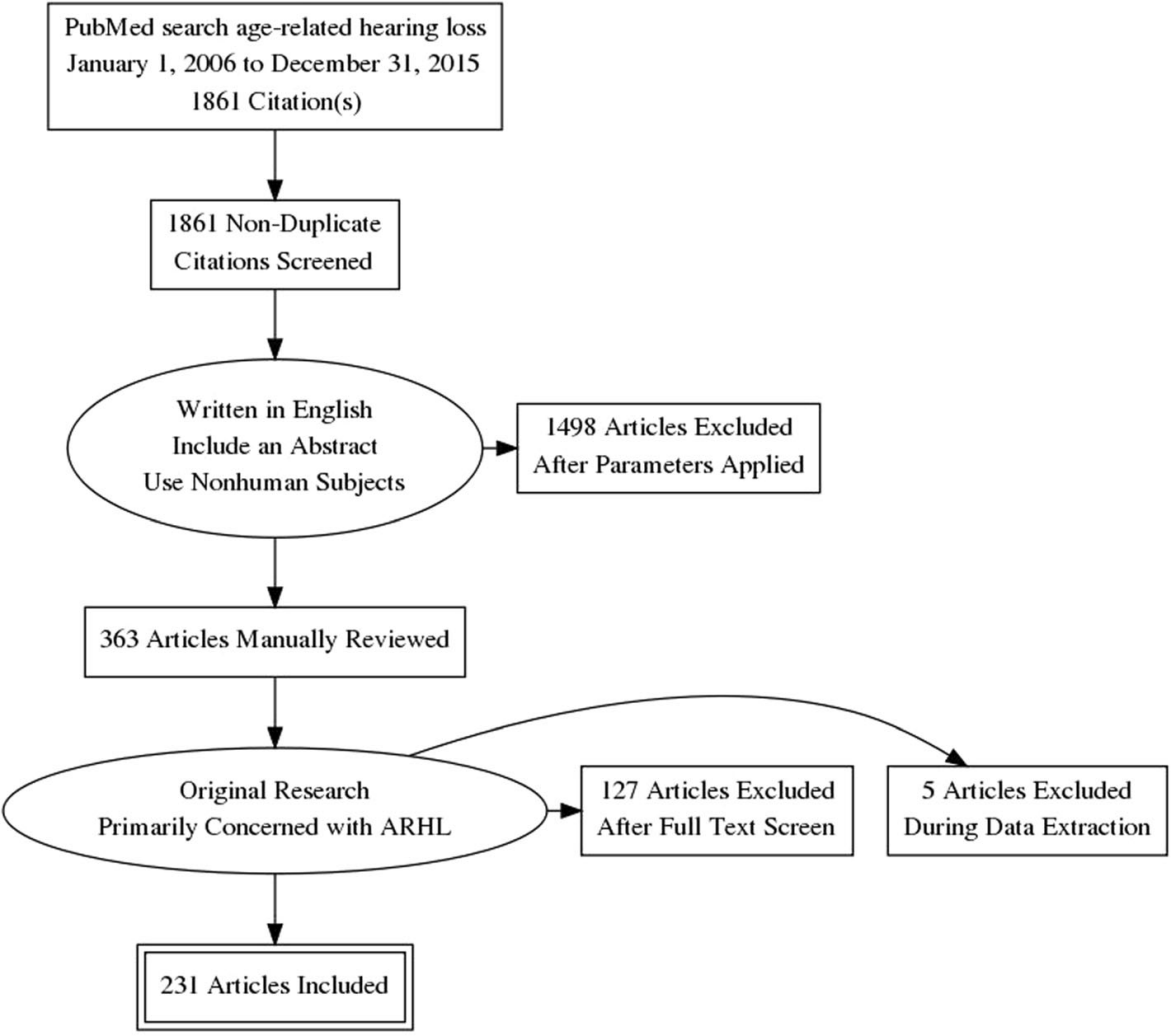
Flow diagram depicting study review method and inclusion criteria

Manuscripts were identified in PubMed using the query “age-related hearing loss,” which generated a total of 4090 unique manuscripts. A parameter for the 10-year period from January 1, 2006, to December 31, 2015, was applied, yielding 1861 manuscripts. Filters were applied to select for manuscripts including an abstract, written in English, and including other animals (non-human), which generated 363 manuscripts. Manuscripts were exported to a Microsoft Excel file and manually screened for exclusion if (1) a majority of the experiments and results were not primarily concerning ARHL (*n* = 62); (2) manuscripts were reviews, meta-analyses, or non-original research (*n* = 61); (3) experiments were not performed in animals (*n* = 5); and (4) had no full text available through PubMed or the Johns Hopkins Libraries (*n* = 4). Following exclusion, a total of 231 manuscripts were included in this study for analysis. Inclusion criteria are depicted in the flow diagram (Fig. 1).

### Variables coded

The full text of each manuscript was reviewed for the sex of the animals (males, females, both, or not specified), number of male and female animals, total number of animals, discussion of sex-based results (yes or no), international- or United States (US)-based study, and year of publication.

### Data analysis

We calculated the total number and percentage of manuscripts reporting number of animals used; total number and percentage of manuscripts reporting the sex; total number and percentage of manuscripts using males, females, or both sexes; and total number and percentage of males and females used. We also calculated the total number and percentage of US- and international-based studies, total number and percentage of US and international studies reporting sex, and total number and percentage of international and US-based studies using only males, only females, or both sexes. We also calculated total number and percentage of studies discussing or not discussing sex-based results, total number of international and US-based studies discussing sex-based results, and percentage of manuscripts discussing sex-based results for the 10-year period.

## Results

### Overall sex bias

**Fig. 2:**
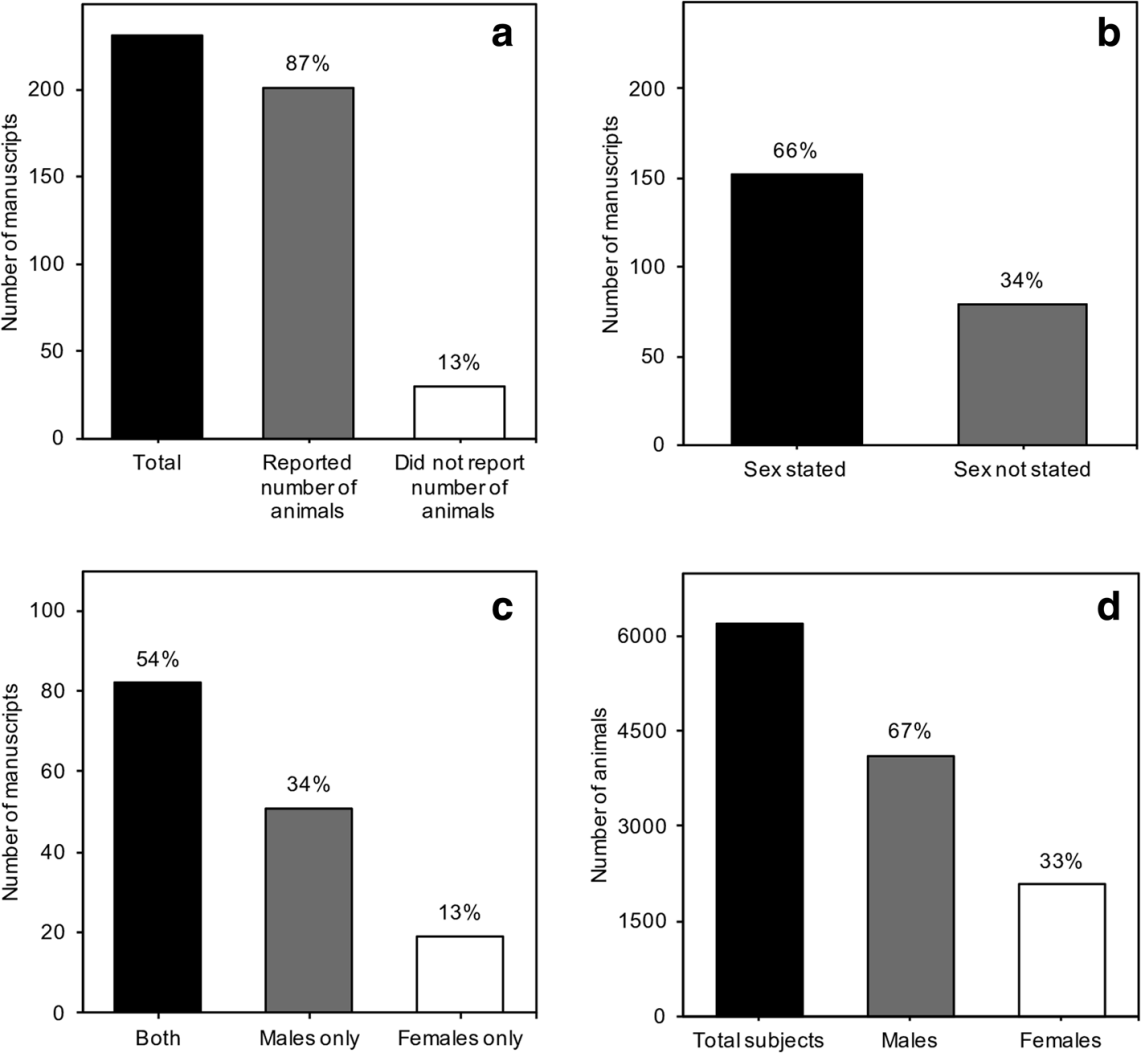
Overall sex bias in preclinical and basic age-related hearing loss research. **a** Total number of manuscripts compared to the number of manuscripts reporting and not reporting total number of animals. **b** Number of manuscripts specifying the sex and not specifying the sex of animals used in the experiments. **c** Number of manuscripts using both sexes, males only, or females only. **d** Total number of animals in all manuscripts reviewed compared to total number of males and females used

Of the total number of manuscripts included, 201 (87%) reported the total number of animals, while 30 (13%) did not (Fig. 2a). One hundred fifty-two (66%) manuscripts reported the sex of animals used in the experiments, while 79 (34%) did not (Fig. 2b). Of these 152 manuscripts reporting sex, 82 (54%) used both sexes, 51 (34%) used males only, and 19 (13%) used females only (Fig. 2c). Of the 154 studies reporting the total number of males (79 studies) and females (46 studies) used, 4125 were males (67%) and 2077 (33%) were females (Fig. 2d). The average number of males per study was 52.2, whereas the average number of females used per study was 45.2.

### Sex bias in US and international studies

**Fig. 3:**
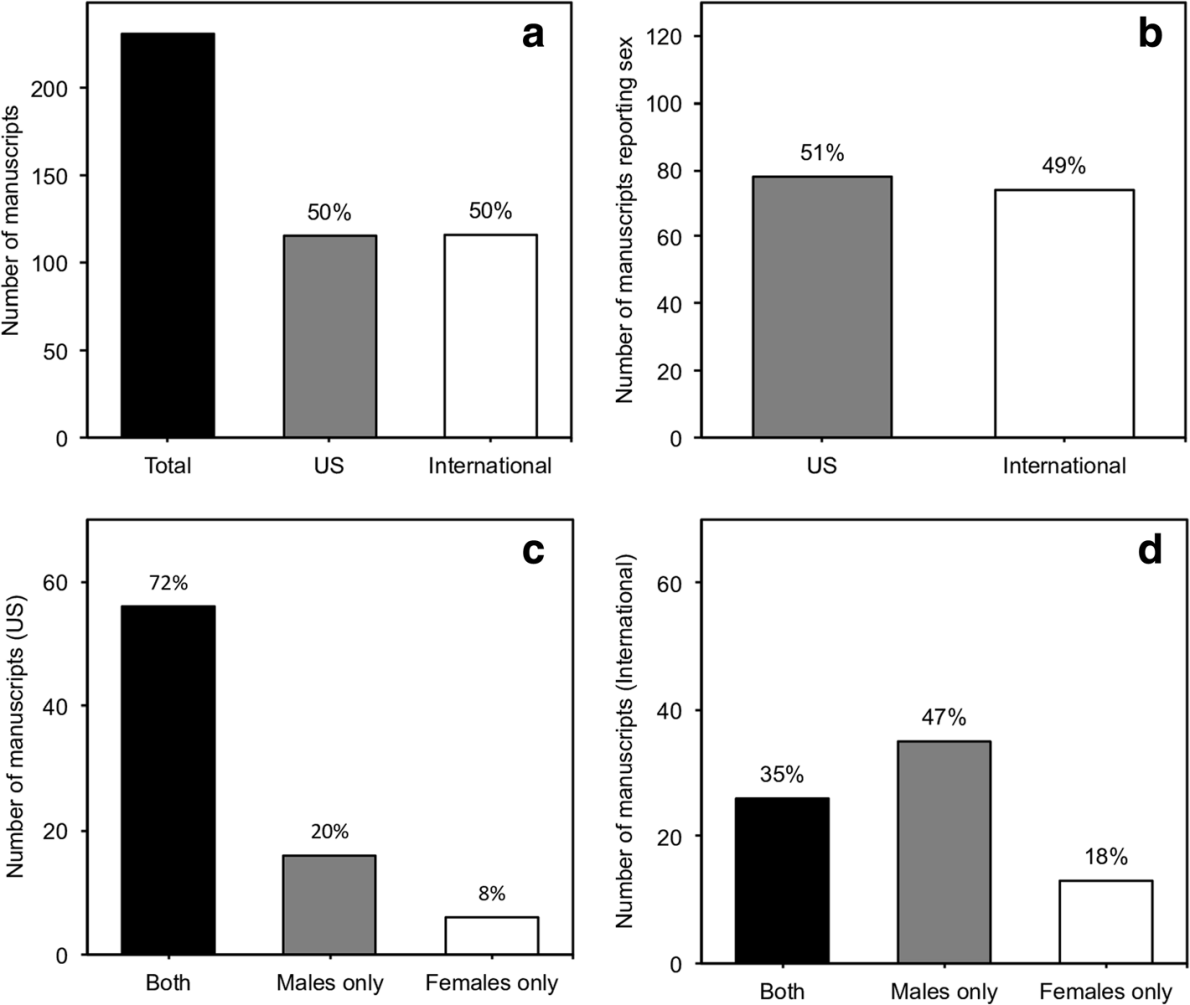
Sex bias in US and international preclinical and basic age-related hearing loss research. **a** Total number of studies compared to the number of studies at US- and international-based sites. **b** Number of US- and international-based studies reporting sex. **c** Number of US-based studies using both sexes, males only, or females only. **d** Number of international-based studies using both sexes, males only, or females only

Of the total number of studies, 115 (50%) were US based and 116 (50%) were internationally based (Fig. 3a). Of the 152 studies reporting sex, 78 (51%) were US based and 74 (49%) were internationally based (Fig. 3b). Of the 78 studies reporting sex based in the US, 56 (72%) used both sexes, 16 (20%) used males only, and 6 (8%) used females only (Fig. 3c). Of the 74 internationally based studies reporting sex, 26 (35%) used both sexes, 35 (47%) used males only, and 13 (18%) used females only (Fig. 3d).

### Sex bias over time

**Fig. 4:**
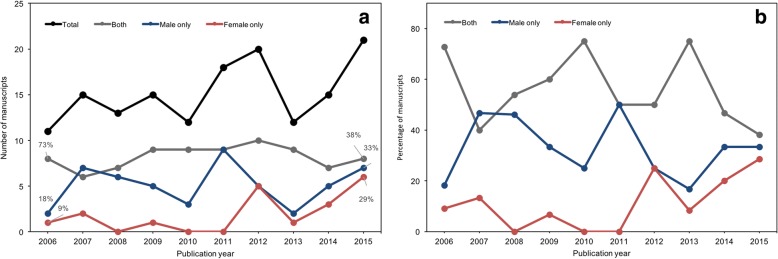
**a** Number and **b** proportion of manuscripts on basic and preclinical age-related hearing loss using both sexes, males only, or females only from 2006 to 2015

Overall, the number of basic and preclinical manuscripts reporting on ARHL increased from a low of 11 in 2006 to a high of 21 in 2015 (Fig. 4a). The percentage of manuscripts including both males and females fluctuated over the 10-year period, but the highest proportion of studies including both sexes was in 2006 (73%) and the lowest proportion of studies including both sexes was in 2015 (38%) (Fig. 4b). The proportion of studies using males only generally increased over the 10-year period from 2006 (18%) to 2015 (33%). 2007 is the only year where a higher proportion of studies included males only (47%) than both sexes (40%). The proportion of studies using females generally increased from 2006 (9%) to 2015 (29%). In no given year did the proportion of studies including only females exceed the proportion of studies using only males or both sexes. In 2008, 2010, and 2011, no studies used females only.

### Reporting of sex-based results

**Fig. 5:**
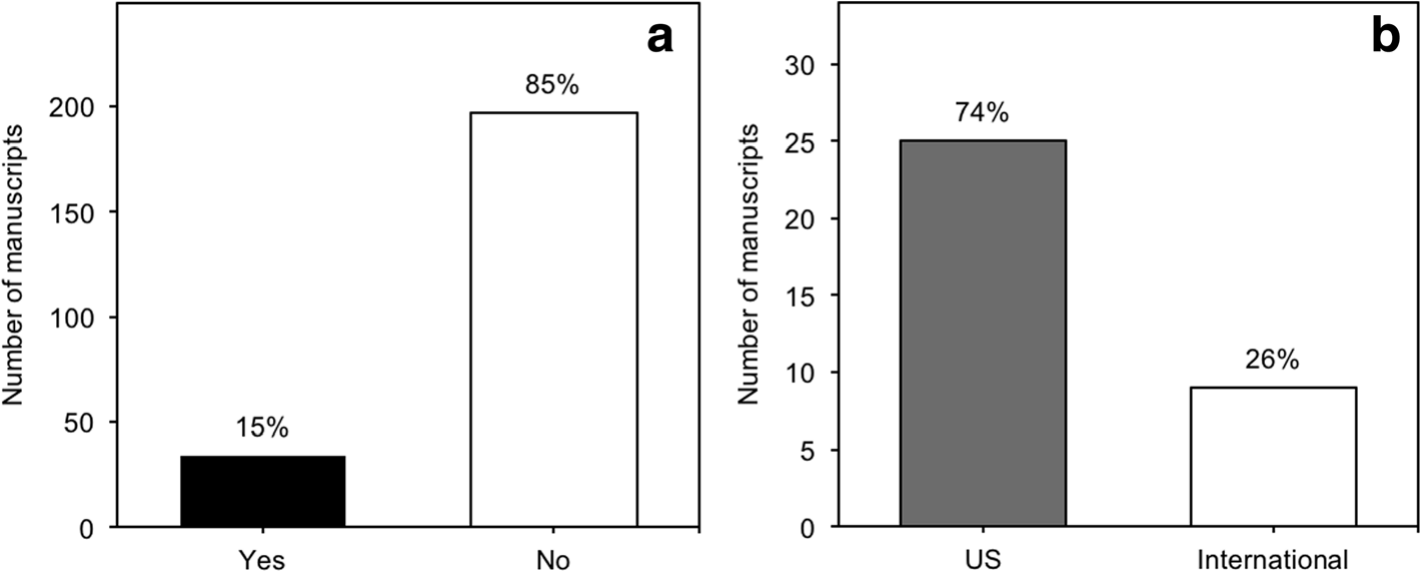
Reporting of sex-based results in preclinical and basic age-related hearing loss. **a** Number of studies reporting sex-based results and studies not discussing sex-based results. **b** Number of US-based and international studies reporting sex-based results

Of the total 231 studies, 34 (15%) reported sex-based results, while 197 (85%) did not (Fig. 5a). Of studies reporting sex-based results, 25 (74%) were US based and 9 (26%) were internationally based (Fig. 5b). Of the 82 manuscripts using both sexes, 24 (29%) reported sex-based results. The percentage of manuscripts reporting sex-based results decreased from a high of 54% in 2006 to a low of 14% in 2015, although the total number of manuscripts on ARHL increased from 11 in 2006 to 21 in 2015.

## Discussion

This study demonstrates sex bias in basic and preclinical ARHL research. Thirteen percent of studies did not report the total number of animals used. About two thirds of papers reported the sex of animals used, and of studies that reported sex, males only were used over 2.5 times more than females only. For studies that reported the sex and total number of animals used, about two thirds of animals used were male and one third were female. There were a nearly equal number of US-based and international-based studies, with about an equal proportion of US and international studies reporting sex. The proportion of studies including both sexes was over two times greater for US-based studies compared to international-based studies. Five times as many papers did not report sex-based results compared to those that did, and of those that did, US-based papers were three times more likely to do so than international-based papers.

Over the 10-year period of 2006 to 2015, the proportion of studies using both sexes of animals decreased by 40%, and whereas the proportion of studies including males only increased nearly twofold, the number of females only increased over threefold. Overall, the number of manuscripts on ARHL increased from 2006 to 2015, but there was a nearly 40% decrease in the number of papers discussing sex-based results. Compared to the previous sex bias study on noise-induced hearing loss (NIHL), this study finds a lower proportion of studies using males only at 34% (compared to 61%) [30]. Additionally, in studies reporting sex and number of animals used, 67% of all animals used in ARHL studies were males, as opposed to the 81% found in the study on NIHL [30].

Some investigators cited increased variability related to estrogen cycles to justify the exclusion of females from ARHL studies [31], yet it has been found that the estrous cycle is not generally an influential factor in differences in variability between male and female rodents [32]. When estrous cycle does affect hearing outcomes, studying these effects will improve our understanding of ARHL and could help identify potential new treatments. For example, studying hearing in women and female animals has led to the recognition of estrogen as a possible protective agent against hearing loss [33].

## Conclusions

Sex bias is prevalent in basic and preclinical ARHL research. The inclusion of both males and females in basic and preclinical ARHL research is critical for translational and clinical research outcomes. If only one sex is tested, it is impossible to know which results will generalize to the other sex. By including both sexes in preclinical research, it should be possible to avoid sex-specific issues later in drug development and clinical studies, such as ineffectiveness or adverse side effects. When treatments and therapies move to a clinical level, it is important to be able to predict any sex-specific outcomes for better personalization of healthcare treatment. Including sex as a biological variable in basic and preclinical research may reveal other mechanisms of sex differences in ARHL and hasten translation to viable prevention and treatment options.

## Abbreviations

ARHL: Age-related hearing loss
NIHL: Noise-induced hearing loss
